# Infection Kinetics and Tropism of *Borrelia burgdorferi* sensu lato in Mouse After Natural (via Ticks) or Artificial (Needle) Infection Depends on the Bacterial Strain

**DOI:** 10.3389/fmicb.2018.01722

**Published:** 2018-07-31

**Authors:** Natacha Sertour, Violaine Cotté, Martine Garnier, Laurence Malandrin, Elisabeth Ferquel, Valérie Choumet

**Affiliations:** ^1^CNR des Borrelia, Institut Pasteur, Paris, France; ^2^BIOEPAR, INRA, Oniris, Université Bretagne Loire, Nantes, France; ^3^Unité Environnement et Risques Infectieux, Institut Pasteur, Paris, France

**Keywords:** lyme borreliosis, *Borrelia*, *Ixodes ricinus*, transmission, tick bite, mouse

## Abstract

*Borrelia burgdorferi* sl is a complex of pathogen bacteria transmitted to the host by *Ixodes* ticks. European *Ixodes ricinus* ticks transmit different *B. burgdorferi* species, pathogenic to human. Bacteria are principally present in unfed tick midgut, then migrate to salivary glands during blood meal and infect a new host via saliva. In this study, efficiency of transmission in a mouse model of three pathogen species belonging to the *B. burgdorferi* sl complex, *B. burgdorferi* sensu stricto (B31, N40, and BRE-13), *B. afzelii* (IBS-5), and *B. bavariensis* (PBi) is examined in order to evaluate infection risk after tick bite. We compared the dissemination of the *Borrelia* species in mice after tick bite and needle injection. Location in the ticks and transmission to mice were also determined for the three species by following infection kinetics. After inoculation, we found a significant prevalence in the brain for PBi and BRE-13, in the heart, for PBi, in the skin where B31 was more prevalent than PBi and in the ankle where both B31 and N40 were more present than PBi. After tick bite, statistical analyses showed that BRE-13 was more prevalent than N40 in the brain, in the bladder and in the inguinal lymph node. When *Borrelia* dissemination was compared after inoculation and tick bite, we observed heart infection only after tick inoculation of BRE-13, and PBi was only detected after tick bite in the skin. For N40, a higher number of positive organs was found after inoculation compared to tick bite. All European *B. burgdorferi* sl strains studied were detected in female salivary glands before blood meal and infected mice within 24 h of tick bite. Moreover, *Borrelia*-infected nymphs were able to infect mice as early as 12 h of tick attachment. Our study shows the need to remove ticks as early as possible after attachment. Moreover, *Borrelia* tropism varied according to the strain as well as between ticks bite and needle inoculation, confirming the association between some strains and clinical manifestation of Lyme borreliosis, as well as the role played by tick saliva in the efficiency of *Borrelia* infection and dissemination in vertebrates.

## Introduction

Lyme borreliosis (LB) is the most commonly occurring vector-borne disease in Europe. It is caused by spirochetes belonging to the *Borrelia burgdorferi* sensu lato (sl) complex. This complex comprises species: *Borrelia burgdorferi* sensu stricto (ss), *B. afzelii, B. garinii, B. bavariensis, B. spielmanii, B. valaisiana* and *B. lusitaniae* (Rijpkema et al., 1997; Collares-Pereira et al., 2004; Rudenko et al., 2011; Stanek et al., 2011). The bacteria are transmitted by a hard tick bite of *Ixodes* genus. Ticks can parasitize a wide range of hosts. Some of them may be reservoirs for *Borrelia*, humans being considered as accidental hosts.

In Europe, nearly 85,000 cases are reported each year, an underestimated figure due to the many undeclared or undiagnosed cases. The highest number of cases is reported in Germany, Austria, the Czech Republic, Slovenia and the Balkans (Lindgren and Jaenson, 2006). In France, the LB incidence rate is around 42 per 100,000 inhabitants (Vandenesch et al., 2014). LB is contracted by both forest professionals and recreational visitors.

Lyme borreliosis (LB) can affect a wide range of tissues including skin, the nervous system, joints, heart, and less frequently other organs (Stanek and Reiter, 2011). The most common presenting symptom is erythema migrans, a typical skin lesion that generally appears within 3–30 days subsequently to the infectious tick bite. Days to weeks after infection, *B. burgdorferi* disseminates through the bloodstream and/or lymphatic system to invade and colonize various tissues, such as the synovial fluid of joints, the heart and the nervous system. Neuroborreliosis is the most common manifestation in Europe and is due to the geographic spread of *B. garinii* (Stanek and Reiter, 2011; Koedel et al., 2015). Arthritis, a late manifestation targeting large joints, is commonly reported in North America, where *B. burgdorferi* ss species are dominant. Cardiac manifestations have been reported during both the acute phase and the chronic stage, but is rare (<5%). Acrodermatitis chronica atrophicans is associated with *Borrelia afzelii* (Coipan et al., 2016).

The epidemiological chain of LB comprises three links: natural reservoirs (small mammals, birds), the vector and animal species known to amplify the tick cycle (large mammals). In Europe, the vector of *B. burgdorferi* sl is the hard tick *Ixodes ricinus*. This tick is present especially in humid and forest areas. It is a mandatory bloodsucking parasite, with a development cycle implying three stages (larva, nymph, adult) interspersed with bloodmeals and molts.

*I. ricinus* is able to feed on more than 240 different species. Reservoirs provide both host and prolonged survival of the pathogen, which increases the likelihood that a tick contracts the bacteria from gorging on the host (Skotarczak, 2009). For most tick-borne pathogens, transmission to the vertebrate host occurs during a blood meal via the saliva of the vector. Both salivary glands and saliva play an important role in the transmission process. Tick saliva contains a large variety of components able to counteract host haemostasis and play a role on the tuning of host immune responses (Šimo et al., 2017). Enhancement of pathogen transmission by tick saliva has been reported for several tick-pathogen associations (Labuda and Nuttall, 2004). Interestingly, the mode of inoculation of *B. burgdorferi* sl was shown to influence infection in inbred strains of mouse (Gern et al., 1993).

Humans are considered accidental hosts and transmission can occur when in contact with a vector-adapted environment, where vertebrate hosts and their associated ticks are present, or when animals transport ticks into areas of human dwelling. It is usually specified in health agency guidelines that the risk of LB rises with duration of attachment of the tick. In the same way, it is regularly specified that the danger of transmission is very low or absent if the tick is removed within 24–48 h (Eisen, 2018).

In this study, we were interested in defining kinetics of mouse infection by ticks (nymphs and adults) infected by different *Borrelia* strains or species. We also compared the dissemination of various *Borrelia* strains and species in mice infected by various modes of inoculation (via infected ticks or by needle injection).

## Materials and methods

### Mice

Specific-pathogen-free mice C3H/HeN (5–7-week-old females) were purchased from Janvier (St Berthevin, France). Mice were housed in the Institut Pasteur's Animal facilities, accredited by the French Ministry of Agriculture to perform experiments on live mice, in appliance with the French and European regulations on care and protection of Laboratory animals. The protocol of our experiments was approved by the Animal Ethics Committee of Pasteur Institute (CETEA-Institut Pasteur) and by the French Ministry of Higher Education and Research (MESR 00762.02).

### Bacterial strains

Table 1 listed the *B. burgdorferi* sensu lato (sl) strains used in this study. They were all cultivated in BSK medium (Sigma) at 33°C. The number and motility of spirochetes were determined by dark-field microscopy with a Petroff Hausser counting chamber (Hausser scientific). The presence of *ospC* was verified by PCR using the following primers: forward BRE-13, B31 and N40: AAAAAAAAGGATCCGGAAAAGATGGGAATGC; forward PBi: AAAAAAAAGGATCCGGTGGGGATTCTGCATC; forward IBS-5: AAAAAAAAGGATCCGGGAAAGGTGGGTC; reverse for all strains and species: AAAAAAAACTCGAGCTAAGGTTTTTTTGGACTTCT TGC.

**Table 1:** Origins of the different *B. burgdorferi* sl strains used in this study.

**Name**	**Species**	**Origin**	**Source**	**OspC groups**	**Preferential tropism**	**Localization**	**References**
B31	*B. burgdorferi* ss	North America	*I. scapularis*	A (Invasive)	Joint	Europe and North America	Fraser et al., 1997
BRE-13	*B. burgdorferi* ss	France	Human (CSF)	Q (invasive)	Joint	Europe	Lagal et al., 2003
PBi	*B. bavariensis*	Germany	Human (CSF)	G4 (Invasive)	Nervous system	Europe	Margos et al., 2009
IBS-5	*B. afzelii*	France	Human (EM)	Invasive	Skin	Europe	–
N40	*B. burgdorferi* ss	North America	*I. scapularis*	E (invasive)	Skin	North America	Pachner and Itano, 1990

*EM, erythema migrans; CSF, cerebrospinal fluid*.

### Infection of ticks

*I. ricinus* nymphs and larvae were obtained from the pathogen-free breeding colony at the UMR BioEpAR (Oniris, France). Ticks were reared and maintained in chambers with a relative humidity of 80–90% at room temperature before feeding.

Each cohort (200 ticks) was controlled for the absence of *B. burgdorferi* sl DNA by PCR.

**Figure 1:**
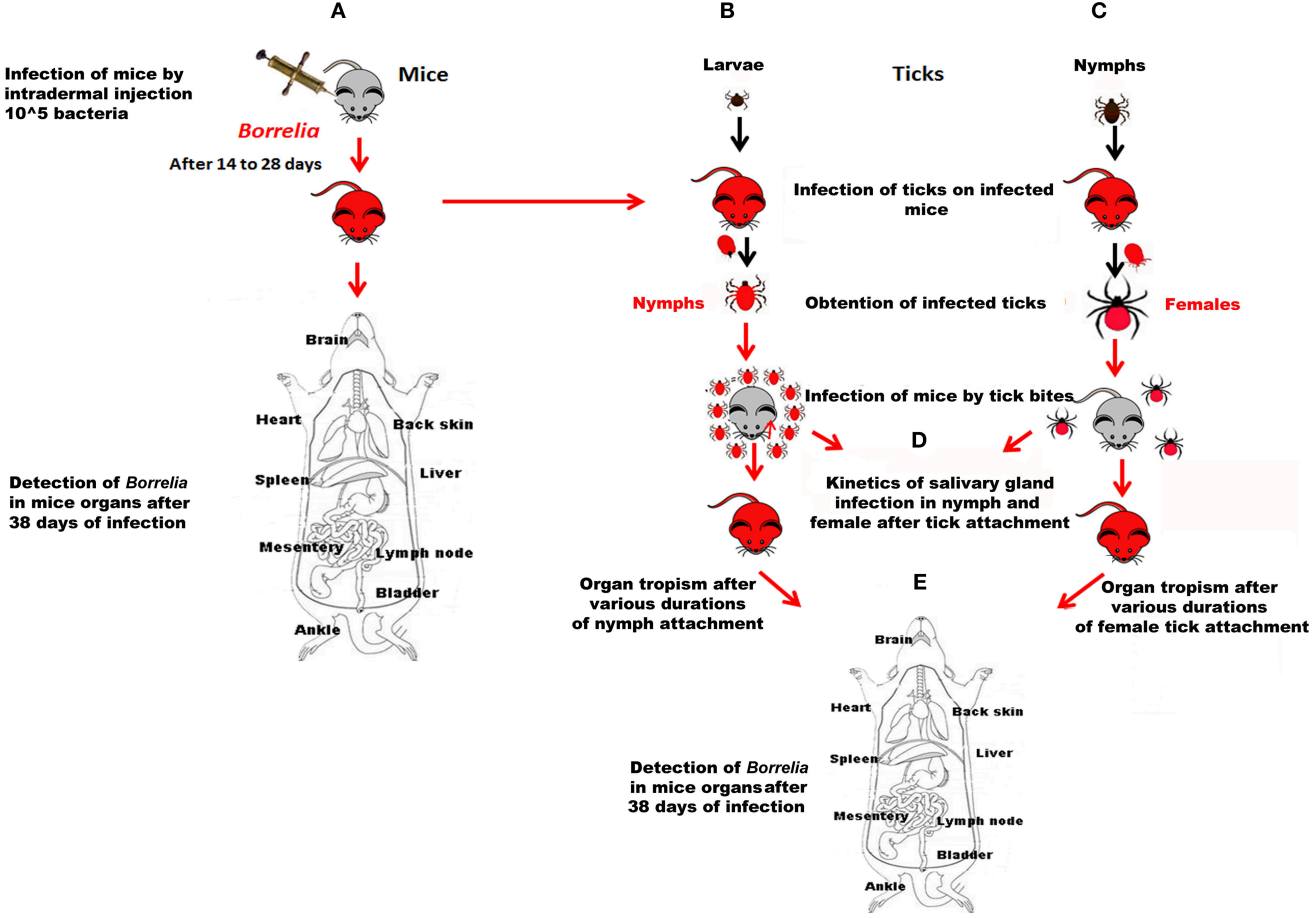
Data acquisition workflow. **(A)** Mice were infected by subcutaneous inoculation of 10^5^ bacteria. Their infection status was controlled by PCR after ear biopsy. *Borrelia*-positive mice were either used for infecting larvae or nymphs (**B,C** respectively) or dissected 38 days after infection; B and C. *Borrelia*-negative larvae or nymphs were fed on *Borrelia*-positive mice. After molting, their rate of infection was determined; **(D)** Ten *Borrelia*-exposed nymphs or 3 *Borrelia*-exposed females were fed on *Borrelia*-negative mice. The kinetics of infection of their salivary glands was determined after different duration of tick attachment; **(E)** Mice exposed to the bite of *Borrelia*-exposed nymphs or female ticks for different durations of tick attachment were dissected 38 days after tick bite.

To obtain infected nymphs or adults, larvae and nymphs were allowed to feed on infected mice until repletion 14–28 days after mouse infection, depending on the result of ear biopsies (Figure 1). For each strain, 2 series of 40 larvae or 20 nymphs were allowed to feed per mouse at 1 week interval. Replete ticks were maintained at 20°C and 95% relative humidity and used about 2 months after they molted into potentially infected nymphs and adults. Tick infection rates were evaluated by DNA detection on the carcasses of 30 nymphs and on a range of 23–36 adults depending on the *Borrelia* strain.

### Infection of mice with *B. burgdorferi* sl

1. Infection of mice by subcutaneous inoculation (Figure 1A). For each strain, ten mice were subcutaneously inoculated with 0.1 ml of the culture material (10^5^ spirochetes). Mice from the control group were injected at the same time with an equal volume of BSK medium.
2. Infection of mice by infected tick-bite (Figures 1B,C). The nymphs and females that were used in all these experiments were fed on *Borrelia*-infected mice at the preceding stage. The back of mice was shaved and a hollow plastic cap was glued with wax (Mbow et al., 1994). Ticks were placed in the plastic cap that is closed by a plaster. They were observed twice a day.
  - Tropism of the various *Borrelia* strains inoculated by tick bites (comparison with needle inoculation of the various strains). Three females were placed in the plastic cap. Five to seven mice were infected up to 5 days [120 h] of female tick attachment.
  - Kinetics of infection of tick salivary glands and of transmission of the different strains after infected tick bite (Figures 1D,E). It was analyzed using ten nymphs or 2–3 females that were allowed to feed on 3 healthy mice for each time of the kinetics. Experiments were repeated 2 or 3 times. The duration of infection was controlled by removing ticks at 12, 24, and 36 h of feeding for nymphs, and at 24, 72, 120 and repletion for adults. The presence of spirochetes in tick salivary glands and in tick organs was analyzed by PCR.
3. Detection of *Borrelia* in mice infected by inoculation or tick bite (Figures 1A,E). To determine *Borrelia* infection in mice, ear biopsies were collected each 7 days after 2 weeks of infection until *Borrelia* DNA detection. After 38 days of *B. burgdorferi* sl inoculation or tick bite infection (the timing started as soon as the tick was attached), mice were dissected and spirochete DNA presence was investigated from different organs (brain, heart, back skin, ankle, bladder, muscle, spleen, liver, mesentery and inguinal lymph nodes), collected aseptically.

### Dissections

Tick dissections were performed under microscope in sterile PBS. All dissection materials were cleaned with alcohol 70% and rinsed with sterile water between each sample. Individual pairs of tick salivary glands, the remaining tick carcasses and all organs from mice were analyzed immediately or frozen at −80°C until DNA extraction.

### *B. burgdorferi* sl DNA detection in ticks and mice

DNA extraction from ticks or mice was performed using the DNeasy Blood and Tissue Kit (Qiagen, Hilden, Germany). The purified DNA was eluted with sterile water in 20 μl for salivary gland, 50 μl for nymph carcasses, 100 μl for adult carcasses and between 100 and 200 μl for each mouse organ.

A nested PCR was performed on each sample to amplify the variable spacer region between two tandemly duplicated genes encoding for ribosomal 23 and 5S. The first set of primers used was Ins1 (5'-GAAAAGAGGAAACACCTGTT-3') and S23R (5′-TCGGTAATCTTGGGATCAAT-3′) which amplified a 360-bp fragment. The second amplification was performed with RRC (5′-CTGCGAGTTCGCGGGAGAG-3′) and RRB (5′-AAGCTCCTAGGCATTCACCATA-3′) primers resulting in a 257-bp fragment (Schwartz et al., 1992).

For each PCR, 5 μl of DNA from the extract or from the first amplification were used. The PCR cycles were carried out with an initial denaturation step for 4 min at 94°C; 35 cycles of denaturation for 1 min at 94°C, annealing for 1 min at 55°C for Ins1-S23R and 59°C for RRB'-RRC', and extension for 1 min at 72°C; and a final extension step at 72°C for 10 min.

Each reaction was conducted in a total volume of 25 μl with 0.3 μmol/μl of each primer, 200 μmol/L of each dNTP, 2.5 μl of 10× PCR buffer, and 1.25 U of *Taq* DNA polymerase (Taq CORE Kits, Q-biogene). All PCRs were performed in a thermocycler My cycler (Bio-Rad, France). Positive control (*B. burgdorferi* sl strain different from the strain used for infection) and negative control (PCR mix) were used in each PCR.

PCR products were electrophoresed in a 2% agarose gel with ethidium bromide and visualized with a Gel Doc™ XR (Bio-Rad).

### Statistical analysis

All statistical analyses were done in Simstat (Provalis Research) using the non-parametric tests Kruskal-Wallis and Mann-Whitney, taking into account the Bonferroni correction.

## Results

### Infection of mice after *B. burgdorferi* sl needle inoculation

All DNA extraction from ear biopsies of mice infected by *B. burgdorferi* ss B31 strain allowed identification of *B. burgdorferi* sl DNA after 21 days of infection. For *B. burgdorferi* ss BRE-13 strain, *Borrelia* DNA was detected in ear biopsies from 20% of mice after 14 days and 70% of mice after 21 days. 90% of mice were found positive by specific DNA amplification 14 days after inoculation of the *B. burgdorferi* ss N40 strain. DNA *B. burgdorferi* sl was detected in the ears of mice infected with *B. bavariensis* PBi strain after 3 weeks in 60% of them and in 80% of mice after 28 days. *Borrelia* DNA was amplified in 40% of mice 2 weeks after *B. afzelii* IBS-5 inoculation and 80% of them were positive 1 week later. Thirty eight days after needle inoculation, all mice were found positive for all *Borrelia* strains.

**Figure 2:**
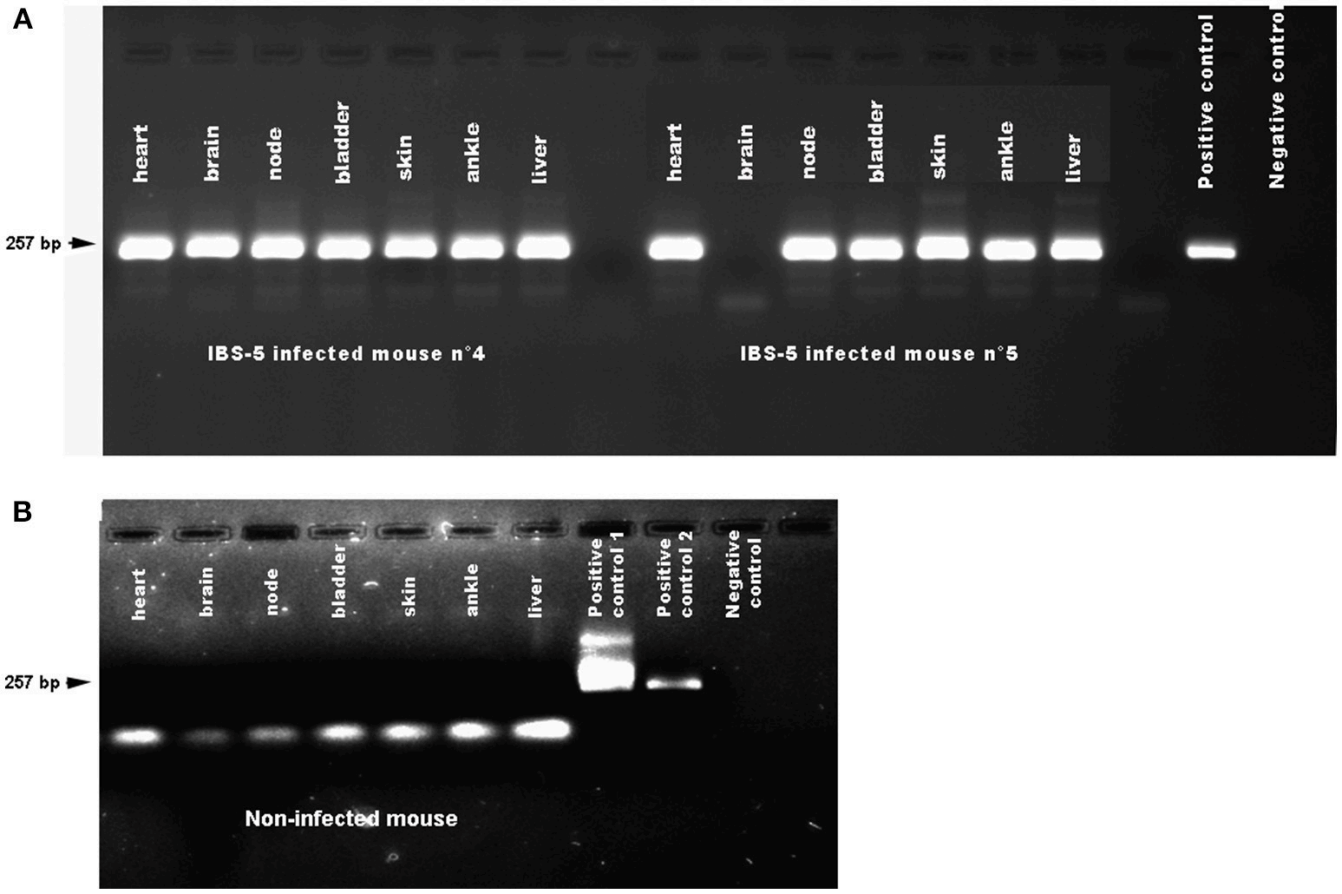
Detection of *Borrelia afzelii* IBS-5 by nested PCR in the organs of mice after needle inoculation. Mice were infected by subcutaneous inoculation of 10^5^ bacteria. Organs were dissected at 38 days post-infection. DNA was extracted as described in the Methods. A first step of PCR was performed followed by a second step of nested PCR. Negative controls (PCR mix) and positive controls (DNA extracted from a culture of *Borrelia burgdorferi* sl bacteria) were used on each gel. **(A)** Organs of mice infected by *B. afzelii* IBS-5. The positive control was a DNA extracted from *B. garinii* 20047 strain. **(B)** Organs of a non-infected mouse. Two positive controls were used. Positive control n°1: *B. garinii* 20047 strain amplified in the first PCR. Positive control 2: *B. garinii* 20047 strain; Negative control: PCR mix.

For the 5 strains, ten organs were studied in order to detect *B. burgdorferi* sl infection by nested PCR (Table 2, Figure 2). If we consider the three strains of *B. burgdorferi* ss, B31, BRE-13 and N40, specific bacterial amplification was obtained from bladder, back skin and ankle. No *B. burgdorferi* sl DNA was detected in brain 38 days after inoculation of B31 and N40 strains. BRE-13 strain was the only one for which DNA amplification was observed in the brain while B31 was the only strain of *B. burgdorferi* ss to be found in the heart.

**Table 2:** Tropism of *B. burgdorferi* sl DNA in mice organs by two modes of infection (inoculation by needle vs. 120 h female tick attachment) analyzed 38 days post-infection.

***B. burgdorferi* sl *s*trains**	**Type of infection**	**Mouse organs**									
		**Brain**	**Heart**	**Back skin**	**Ankle**	**Muscle**	**Spleen**	**Liver**	**Bladder**	**Mesentery**	**Lymph node**
		**% of organ infected**	**% of organ infected**	**% of organ infected**	**% of organ infected**	**% of organ infected**	**% of organ infected**	**% of organ infected**	**% of organ infected**	**% of organ infected**	**% of organ infected**
B31	Inoculation	0^*^	40	**100**^a^^$^	**100**^c^^£^	ND	ND	ND	80	ND	ND
	Tick bite	40	40	40	40	40	60	20	80	40	20
BRE-13	Inoculation	83^*^	0^§^	67	50	ND	ND	ND	100	ND	ND
	Tick bite	**85**^+^	57^b^	85	71	40	60	40	**85**^+^	20	**85**^+^
N40	Inoculation	0^*^	0^§^	60	**100**^c^^£^	60^d^	0	0	60^d^	60^d^	100^e^
	Tick bite	0^+^	0	100	14	0	0	0	0^+^	0	0^+^
PBi	Inoculation	**100**^f^^*^	**100**^g^^§^	0^$^	0^£^	ND	ND	ND	67	ND	ND
	Tick bite	14	0	57^b^	43	57	71	71	71	0	43
IBS-5	Inoculation	40	80	80	80	ND	ND	ND	60	ND	ND
	Tick bite	43	28	100	43	28	43	71	43	28	43
Stat	Inoculation	*p* < 0.0003	*p* < 0.0002	*p* < 0.005	*p* < 0.004	–	–	–	NS	–	–
Stat	Tick bite	*p* < 0.018	NS	NS	NS	NS	NS	NS	*p* < 0.016	NS	*p* < 0.017

Mice were infected with the various strains/species of Borrelia burgdorferi sl either by needle injection of 10^5^ Borrelia/mouse or by tick bite during 120 h (see Figure 1). Mice were dissected 38 days after beginning of tick attachment. DNA was extracted as described in the Material and Methods section and a specific nested PCR was performed. The percent of organs found infected in the mice analyzed was reported according to the type of infection (inoculation/tick bite). 5–7 mice were used for each experiment. Statistical comparisons of organ infection between Borrelia strain after infection (inoculation or tick bite) are presented in bold:

* BRE-13 = PBi > B31 = N40;

§ PBi > BRE-13 = N40;

$ B31 > PBi;

£ B31 = N40 > PBi;

+ BRE-13 > N40. Statistical comparison of organ infection between inoculation and tick bite:

a p < 0.04;

b p < 0.03;

c p < 0.004;

d p < 0.017;

e p < 0.0005;

f p < 0.002;

g *p < 0.0003. ND, not done. NS, non-significant*.

After inoculation with *B. bavariensis* PBi strain, bacterial DNA amplification was obtained from bladders, brains and hearts. No bacterial DNA was amplified from back skins and ankles.

Specific DNA was amplified after *B. afzelii* IBS-5 inoculation from hearts, brains, back skins, ankles and bladders.

We then compared the tropism of the various *B. burgdorferi* sl strains after injection. Difference of prevalence was observed in various organs: brain, heart, back skin and ankle. As described above, the three *B. burgdorferi* ss strains did not give similar results. B31 and N40 were never present in the brain at the difference of BRE-13 and PBi (*p* = 0.0003). BRE-13 and N40 were not identified in the heart at the difference of PBi (*p* = 0.0002). B31 and N40 were more prevalent in the ankle than PBi (*p* < 0.004). B31 was found more prevalent than PBi in the back skin (*p* < 0.005). IBS-5 was identified in all examined organs while PBi was not found in the back skin and ankle.

### *B. burgdorferi* sl infection in *I. ricinus* ticks after infection on needle-infected mice

A total of 2,400 nymphs and about 1,500 larvae were used to obtain infected ticks.

On 400 nymphs used for each strain, 224 (56%), 284 (71%), 315 (79%), 233 (58%), and 270 (68%) were engorged and spontaneously detached from mice infected respectively with *B. burgdorferi* ss B31, BRE-13, and N40, *B. bavariensis* PBi and *B. afzelii* IBS-5 strains. After 3 months, respectively 145 (65%), 247 (87%), 255 (81%), 116 (50%), and 261 (97%) nymphs molted into adults from which 79, 93, 120, 45, and 109 females were obtained for each strain.

Female infection rates of the 3 *B. burgdorferi* ss strains reached 70% for B31 (16/23), 78% (28/36) for N40 and 96% for BRE-13 (27/28), the latter strain having the highest rate of infection of the *B. burgdorferi* ss strains (*p* = 0.008). Infection rates in females were 70% (23/33) for *B. bavariensis* PBi and 70% (21/30) for *B. afzelii* IBS-5. When all strains were compared, their rate of infection was similar.

Concerning larvae, 338, 360, and 390 of them were engorged from mice infected respectively with *B. burgdorferi* ss BRE-13, *B. bavariensis* PBi and *B. afzelii* IBS-5 strains. After 3 months, respectively 59, 60, and 61% of BRE-13, *B. bavariensis* PBi and *B. afzelii* IBS-5 engorged larvae molted into nymphs. The infection rates in nymphs were 90% (27/30) for *B. burgdorferi* ss BRE-13, 70% (21/30) for *B. bavariensis* PBi and 77% (23/30) for *B. afzelii* IBS-5.

### *B. burgdorferi* sl tropism in mice after infected tick bite: comparison with needle injection

Mice were infected by ticks that were removed at 120 h of tick attachment. They were sacrificed at 38 days post-infection and their organs dissected. *Borrelia* DNA was detected after nested PCR. The percent of positive organs as function of the strain is reported in Table 2.

After tick bite infection with *B. burgdorferi* ss B31 and BRE-13 strain, all organs presented bacterial DNA amplification with various infection rates while in N40-infected mice, only ankles and back skin were found infected. After bite of *B. bavariensis* PBi infected ticks, bacterial DNA amplification was obtained from all organs except heart and mesentery. *B. burgdorferi* DNA was amplified after bite of *B. afzelii* IBS-5 infected females from all tested organs.

When the prevalence of all strains were compared in each organ, statistical analyses showed that *B. burgdorferi* ss BRE-13 was more prevalent than *B. burgdorferi* ss N40 in the brain (*p* < 0.018), in the bladder (*p* < 0.016) and in the inguinal lymph node (*p* < 0.017) after tick bite.

We compared the dissemination of each strain in the various organs after inoculation or tick bite. We observed the infection of the heart after tick inoculation of *B. burgdorferi* ss BRE-13 whereas this organ was found uninfected after inoculation. The same trend was also observed for *B. bavariensis* PBi for which an infection of the back skin was only detected after tick bite. For the other comparisons, more organs were found infected after inoculation than after tick bite. For instance, a higher number of positive organs was statistically found after inoculation of *B. burgdorferi* ss N40 compared with tick bite.

### Adult tick infection kinetics of salivary glands and mice by *B. burgdorferi* sl after female tick bite

All results of *B. burgdorferi* sl DNA detection in female salivary glands and corresponding mice are reported in Table 3. Mouse infection was detected 38 days after removing infected female ticks.

**Table 3:** Adult tick infection kinetics of salivary glands and mice by *B. burgdorferi* sl after female tick bite.

***B. burgdorferi* sl strains**	**Samples**	**Infection rate %**				
		**Unfed ticks**	**24 h**	**72 h**	**120 h**	**Repletion**
B31	Tick salivary glands N (%)	0/3 (0)^*^	0/6 (0)^$^	ND	4/6 (67)	ND
	Mice (%)	–	0	ND	100	ND
BRE-13	Tick salivary glands N (%)	3/3 (100)^*^	3/3 (100)^$^	6/6 (100)	4/4 (100)	5/5 (100)
	Mice (%)	–	100	100	100	100
N40	Tick salivary glands N (%)	0/3 (0)^*^	2/4 (50)	6/6 (100)	5/6 (83)	4/4 (100)
	Mice (%)	–	100	100	100	100
PBi	Tick salivary glands N (%)	3/3 (100)^*^	3/5 (60)	3/6 (50)	6/6 (100)	ND
	Mice (%)	–	100	100	86	100
IBS-5	Tick salivary glands N (%)	3/3 (100)^*^	2/5 (40)	3/5 (50)	2/4 (50)	4/4 (100)
	Mice (%)	–	100	100	100	100
Stat	Tick salivary glands	*p* < 0.003	*p* < 0.04	NS	NS	NS

Adult female ticks were infected on mice injected with different strains of Borrelia burgdorferi sl. Tick salivary glands were dissected before and after different times of attachment on non-infected mice. Mice exposed to infected tick bites were dissected 38 days after infection. Their rate of infection was determined based on the detection Borrelia DNA in the examined organs (see Figure 1). Two to three ticks were placed on the back of each mouse depending on the time of attachment. After this time, ticks were removed and their salivary glands were dissected and the percentage of Borrelia-positive salivary glands was determined by nested PCR. Statistical comparison of salivary gland infection by the various strains:

* BRE-13 = PBi = IBS5 > B31 = N40;

$ *BRE-13 > B31. ND, not done; NS, non-significant*.

Concerning *B. burgdorferi* ss B31 infected ticks, salivary gland extract DNA showed no *Borrelia* amplification for tick attachment of 24 h. But after 120 h of female feeding, the specific DNA fragment was amplified from 67% of the salivary glands tested. 100% of the mice were found infected for tick attachment of 120 h.

The *B. burgdorferi* ss N40 strain was detected in tick salivary glands collected at 24 h of tick fixation with bacterial DNA amplification from 50% of female salivary glands. At respectively 72 and 120 h of tick attachment, 100 and 83% of salivary glands showed *B. burgdorferi* sl DNA amplification. Furthermore, 100% of mice became infected by N40 strain up to 24 h of tick attachment as well as up to 72 and 120 h of tick bite.

Concerning the 3 other strains, *B. burgdorferi* ss BRE-13, *B. bavariensis* PBi and *B. afzelii* IBS-5, *B. burgdorferi* sl DNA was amplified from 100% of female salivary glands infected at the preceding stage prior to their fixation on mice. From 24 h to repletion, the rate of infection of tick salivary glands with the three different strains was shown to vary depending on the time (Table 3).

When ticks were removed at 24 h of tick attachment, *B. burgdorferi* sl DNA was detected in mice for the three strains: 100 % was infected by *B. burgdorferi* ss BRE-13, *B. bavariensis* PBi and *B. afzelii* IBS-5. This rate of infection was constant after bite of *B. burgdorferi* ss BRE-13 and *B. afzelii* IBS-5 whatever tick attachment time, whereas a slight decrease was observed at 120 h of tick attachment for *B. bavariensis* PBi (Table 3).

We then tested the tropism of the various strains as function of the time of attachment of infected ticks (Table 4). Again, significant differences were found between the various strains of *B. burgdorferi* ss. BRE-13 was the one for which all organs tested except the heart was found infected after 24 h of tick attachment, whereas only the skin and ankle were found infected by N40 and none by B31. Ankles were found positive for all strains except B31 while only BRE-13 and PBi were found in the bladder and in the lymph nodes. The tropism of IBS-5 and N40 was similar.

**Table 4:** Tropism of *B. burgdorferi* sl strains in mice after various durations of female tick attachment.

	**BRE-13**	**PBi**	**IBS-5**	**N40**	**B31**	**BRE-13**	**PBi**	**IBS-5**	**N40**
	**24 h**					**72 h**			
Brain	1/1	0/1	0/1	0/1	0/2	1/1	1/1	0/1	0/1
Heart	0/1	0/1	0/1	0/1	0/2	1/1	0/1	0/1	0/1
Skin	1/1	0/1	1/1	1/1	0/2	0/1	1/1	1/1	1/1
Ankle	1/1	1/1	1/1	1/1	0/2	1/1	1/1	1/1	0/1
Bladder	1/1	1/1	0/1	0/1	0/2	1/1	1/1	0/1	0/1
Lymph node	1/1	1/1	0/1	0/1	0/2	1/1	0/1	1/1	0/1
	**120 h**					**Repletion**			
Brain	6/7	1/7	3/7	0/7	2/5	1/1	0/1	0/1	0/1
Heart	4/7	0/7	2/7	0/7	2/5	0/1	0/1	1/1	0/1
Skin	6/7	4/7	7/7	7/7	2/5	0/1	1/1	1/1	1/1
Ankle	5/7	3/7	3/7	1/7	4/5	1/1	1/1	0/1	1/1
Bladder	6/7	5/7	3/7	0/7	4/5	1/1	1/1	1/1	0/1
Lymph node	6/7	3/7	3/7	0/7	1/5	1/1	0/1	1/1	0/1

*At least three mice were exposed to tick bite for 24 h, 72 h and repletion and ten mice were exposed for 120 h. Some of them were dissected at 38 days post-tick exposition. In each column are presented the number of mice infected (based on the detection of Borrelia DNA by nested PCR in the organs) divided by the number tested*.

After removing the ticks at 72 h, PBi was found in all organs except the lymph node and the heart, this latter was never infected by this strain whatever the time of attachment. PBi was found in the brain at 72 h of tick attachment and was still detected at 120 h but not any more after repletion of the tick. The bladder was found positive for all strains except N40 after removing the ticks at 120 h or after repletion. Interestingly, we found fewer organs infected after repletion of the tick whatever the strain.

### *B. burgdorferi* sl infection kinetics after nymph bites

No *B. burgdorferi* sl DNA was amplified from nymph salivary glands infected at the preceding stage prior to their fixation on mice. Mice were found infected as soon as 12 h of attachment of *B. burgdorferi* ss BRE-13, *B. bavariensis* PBi and *B. afzelii* IBS-5 infected nymphs (Table 5). All organs except lymph node were found positive after removing nymphs at 12 h for BRE-13 whereas only the ankle and lymph node were infected for PBi, and the brain for IBS-5 after the same time of contact with the infected nymphs.

**Table 5:** Kinetics and tropism of *Borrelia* transmission to mice by infected nymphs.

***Borrelia* strain**	**Time of tick attachment (h)**	**Brain**	**Heart**	**Back skin**	**Ankle**	**Muscle**	**Spleen**	**Liver**	**Bladder**	**Mesentery**	**Lymph node**
BRE-13	12	+	+	+	+	+	+	+	+	+	−
	24	+	+	−	−	+	+	−	−	−	+
	36	+	−	+	+	+	+	+	+	−	−
PBi	12	−	−	−	+	−	−	−	−	−	+
IBS-5	12	+	−	−	−	−	−	−	−	−	−

*Ten Borrelia-exposed nymphs were placed in plastic caps on 3 mice for each strain. Nymphs were detached at the indicated time points. Mice were sacrificed 38 days post-infection and Borrelia burgdorferi sl DNA was detected by nested PCR in the indicated organs. +, at least one mouse had a positive organ; −, none of the examined mice presented a positive organ*.

## Discussion

*Borrelia burgdorferi* sl is transmitted to the host during *Ixodes* ticks blood feeding. Several studies have shown the role that tick saliva could play in tick feeding and pathogen transmission. The minimal infected tick attachment time on a vertebrate host for efficient transmission is still a matter of debate (Cook, 2015). Indeed, it is frequently specified that the risk of infection is lower if the tick is removed within 24–48 h (Piesman et al., 1987; des Vignes et al., 2001). An increased risk of infection is observed with longer tick attachment durations. Several studies showed that a blood meal is able to trigger the downregulation of OspA/B (involved in the colonization and survival of the tick midgut) and the upregulation of OspC (involved in the dissemination from the midgut to the salivary glands in the tick, and the interaction with the vertebrate host) (Grimm et al., 2004; Stewart et al., 2006; Fingerle et al., 2007; Kenedy et al., 2012). These processes are suggested to prepare the bacteria for the infection of vertebrate hosts.

However, data from the literature showed that transmission can occur in less than 16 h (Hynote et al., 2012). In most unfed ticks, spirochetes were shown to be present in the midgut and need to migrate during blood feeding to the salivary glands, from which they are transmitted to the host via saliva (Ribeiro et al., 1987; Zung et al., 1989). However, disseminated infection was observed in ticks before feeding (Piesman et al., 1987). Moreover, there is evidence that *Borrelia* transmission and virulence depend on tick and *Borrelia* species (Moskvitina et al., 1995a,b).

In the present study, the efficiency and the kinetics at which *Ixodes ricinus* ticks are able to transmit *B. afzelii, B. bavariensis* and several strains of *B. burgdorferi* ss to mice were examined in relation to blood meal length. We also examined the tropism of these various strains according to the transmission mode: infected tick bite vs. needle inoculation.

B31 and N40 are two widely studied strains of *B. burgdorferi* ss, which belong to two different 16 S-23 S rRNA spacer types and outer surface protein C (OspC) allelic groups. They were both isolated from *Ixodes scapularis* ticks, and both are very infectious in the mouse model (Burgdorfer et al., 1982; Barthold et al., 1988). Although phylogenetic analyses predicted B31 to be more infectious, Chan et al. (Chan et al., 2012) indicated that N40 strain was more infectious at lower doses of inoculation. BRE-13 belongs to another group of OspC and was isolated from the CSF of a patient with neurological symptoms (B. Degeilh, personal communication).

Our observations showed that the two *B. burgdorferi* ss N40 and B31 strains were not present in unfed adult tick salivary glands and that the transmission to mice was observed earlier (24 h) for N40 and from 120 h after tick attachment for B31. This was not what we observed for BRE-13, another strain of *B. burdorferi* ss, for which unfed adult ticks had systemic infections. We thus observed a strain-specific variation in the tissue tropism in ticks. However, since we set the first infection duration at 24 h, we were not able to see any difference in the kinetics of infection in mouse between BRE-13 and N40.

*B. afzelii* IBS-5 and *B. bavariensis* PBi were also present in unfed adult tick salivary glands, as was previously shown for nymphs infected by *B. garinii* and *B. afzelii* (Kahl et al., 1998). In agreement with this observation, mice were found infected as early as 24 h of tick attachment.

For ticks with systemic infection, we did not find that the risk of transmission increased with the contact duration with host since all mice were found infected whatever the duration of attachment. For strains like B31 and N40 that were not found in salivary glands before feeding, we found a difference in the kinetics of infection. B31 has not reached the salivary glands at 24 h after tick attachment while N40 was certainly present in this organ before 24 h and able to infect the mice. We were also able to show that the dissemination of the various strains differed according to the duration of attachment. For instance, BRE-13 was isolated in several organs except the heart after only 24 h of tick attachment, the heart being found infected after 72 h of tick feeding. PBi was only identified in the brain when removing the tick after 72 h but not before. For IBS-5, all organs were found infected only at 120 h of tick attachment. These observations may suggest that the symptomatology and thereby the severity of the infection may be correlated to the duration of attachment, at least for some strains, since BRE-13 seems virulent shortly after tick attachment and N40 infects the same organs whatever the time of feeding.

We also studied the infected nymph attachment duration to obtain infected mice. We did not find any systemic infection in *B. burdorferi* ss BRE-13, *B. bavariensis* PBi and *Borrelia afzelii* IBS-5 infected nymphs. However, mice were infected when nymphs were attached for less than 12 h. Again almost all organs tested, including the brain and the heart are infected after 12 h of BRE-13-infected nymph feeding, while PBi was only detected in the ankle and in the lymph nodes and IBS-was identified in the brain.

The ticks, vertebrate hosts and pathogens co-evolution has led to the development of strategies to use salivary components in order to guarantee both pathogen acquisition and transmission, as well as local and disseminated infection in the host (Šimo et al., 2017). Various studies have shown a higher efficiency in *Borrelia* transmission in presence of saliva or saliva components (Wikel, 2013). The salivary gland protein Salp15 plays an immunosuppressive role and may thereby facilitate infection by the low numbers of spirochetes that are released in the skin during tick feeding (Anguita et al., 2002; Ramamoorthi et al., 2005). A tick antioxidant was shown to facilitate tick acquisition of spirochetes from infected animals (Narasimhan et al., 2007). We were therefore interested in comparing the various *B. burgdorferi* sl strains tropism after needle injection or tick bite. In the murine model, mice can be infected by needle inoculation or tick feeding (Tilly et al., 2008). Most mouse strains, as well as the natural reservoir hosts, display no sign of disease, but develop a serological response to *B. burgdorferi* proteins and a persistent infection (Schwan et al., 1988; Barthold et al., 1990; Wu et al., 2013).

After needle injection, we found a difference of tropism in the brain, in the heart, in the back skin and in the ankle for the different strains tested. *B. burdorferi* ss BRE-13 and *B. bavariensis* PBi were detected in the brain of mice while *B. burdorferi* ss B31 and N40 were never detected. The *B. burgdorferi* ss strains that we used in this study were shown to present differential tropism. They belong to different OspC groups (Table 1). OspC is known to be involved in the early stages of mouse infection as well as in dissemination (Kenedy et al., 2012). We can therefore hypothesize that this could explain the altered dissemination observed in the brain for B31 and N40 compared to BRE-13. Interestingly, Wu et al. showed that *B. burdorferi* ss B31 infected mouse brain but it was after intraperitoneal injection of the spirochetes (Wu et al., 2013) suggesting that the intraperitoneal vs. subcutaneous injection route may affect *Borrelia* bacteria tropism. Moreover, the strain of the mice used in our studies are not the same, Balb/C vs. C3H/HeN, this latter being the most relevant animal model for Lyme borreliosis for discriminating infectivity and pathogenicity of various *B. burgdorferi* strains (Chan et al., 2012). However, in agreement with Wu et al. (2013) we were also able to show that statistical differences existed between the tropism of B31 and PBi (*B. bavariensis* but formerly *B. garinii*) in the brain and in the heart.

A significantly higher number of ankles positive for *B. burdorferi* ss B31, BRE-13 and N40 was observed compared with *B. bavariensis* PBi. These results are in agreement with the association between some clinical manifestations of LB and the species of *B. burgdorferi* sl. Arthritis was shown to be caused prevalently by *B. burgdorferi* ss, and neuroborreliosis by *B. burgdorferi* ss and *B. garinii*/*B. bavariensis* (Balmelli and Piffaretti, 1995).

All organs were found positive after feeding with ticks infected with *Borrelia burgdorferi* ss B31 and BRE-13 and with *Borrelia afzelii* IBS-5. Interestingly, all strains except N40 were neurotropic after 120 h of tick feeding.

When we compared the tropism of the various *Borrelia* species between tick bite and needle inoculation, it appears that most of the time the percentage of positive organs was higher after inoculation, except for *B. burdorferi* ss BRE-13 that was found in the heart and for *B. bavariensis* PBi, which is detected in the back skin only after tick bite. These two examples could illustrate the role of tick saliva in *Borrelia* dissemination and tropism. N40 was the strain for which the highest number of positive organs was observed after needle injection while it was detected in only two organs after tick bite: the back skin and the ankle. These results suggest that the number of *Borrelia* injected by needle (10^5^) may exceed by far what is injected by the tick. When *Borrelia* bacteria were quantified by Q-PCR in salivary glands of *Ixodes ricinus* infected by different strains, their level was shown to differ according to the strain (Cotté et al., 2014). The highest level was found in *B. burdorferi* ss BRE-13-infected salivary glands (10^6^), while 10^4^ bacteria were detected in *B. burdorferi* ss B31 and *B. bavariensis* PBi infected glands, the lowest amount, 10^3^, being found in *B. afzelii* IBS-5 infected salivary glands.

In conclusion, we showed that salivary glands of adult ticks infected by the strains *B. burdorferi* ss BRE-13, *B. burdorferi* ss PBi and *B. afzelii* IBS-5 are infected before feeding. The spirochetes are thus present in the salivary glands, thereby potentially able to be injected immediately after tick attachment to host. One hundred percent of mice were found infected as early as 24 h of tick attachment. Interestingly, in contradiction with other studies, we did not report any increase in mice infection rate depending on feeding time, while it is observed for *B. burdorferi* ss B31. We also showed that even though the salivary glands of unfed nymphs infected by *B. burgdorferi* ss BRE-13 *B. bavariensis* PBi and *B. afzelii* IBS-5 are not infected, *Borrelia* transmission was shown to occur as early as 12 h of tick attachment. It is therefore important to remove nymphs or adult ticks as soon as possible after bite to prevent any transmission.

*Borrelia* bacteria tropism tested in this study was shown to vary according to the strain as well as between ticks bite and needle inoculation. These results confirm the association between some strains and clinical manifestation of the LB, as well as the role played by tick saliva in the efficiency of *Borrelia* bacterial infection and dissemination in vertebrates.

## Author contributions

VCH and EF designed and analyzed the experiments. NS, VCO, and MG performed and analyzed the experiments. LM provided the ticks for the experiments. VCH, EF, and LM wrote the manuscript.

### Conflict of interest statement

The authors declare that the research was conducted in the absence of any commercial or financial relationships that could be construed as a potential conflict of interest.

## References

[B1] Anguita J. Ramamoorthi N. Hovius J. W. Das S. Thomas V. Persinski R.. (2002). Salp15, an *Ixodes scapularis* salivary protein, inhibits CD4^+^ T cell activation. Immunity 16, 849–859. 10.1016/S1074-7613(02)00325-4 12121666

[B2] Balmelli T. Piffaretti J. C. (1995). Association between different clinical manifestations of Lyme disease and different species of *Borrelia burgdorferi* sensu lato. Res. Microbiol. 146, 329–340. 7569327 10.1016/0923-2508(96)81056-4

[B3] Barthold S. W. Beck D. S. Hansen G. M. Terwilliger G. A. Moody K. D. (1990). Lyme borreliosis in selected strains and ages of laboratory mice. J. Infect. Dis. 162, 133–138. 2141344 10.1093/infdis/162.1.133

[B4] Barthold S. W. Moody K. D. Terwilliger G. A. Jacoby R. O. Steere A. C. (1988). An animal model for Lyme arthritis. Ann. N Y Acad. Sci. 539, 264–273. 3263827 10.1111/j.1749-6632.1988.tb31860.x

[B5] Burgdorfer W. Barbour A. G. Hayes S. F. Benach J. L. Grunwaldt E. Davis J. P. (1982). Lyme disease-a tick-borne spirochetosis? Science 216, 1317–1319. 7043737 10.1126/science.7043737

[B6] Chan K. Awan M. Barthold S. W. Parveen N. (2012). Comparative molecular analyses of *Borrelia burgdorferi* sensu stricto strains B31 and N40D10/E9 and determination of their pathogenicity. BMC Microbiol. 12:157. 10.1186/1471-2180-12-157 22846633 PMC3511255

[B7] Coipan E. C. Jahfari S. Fonville M. Oei G. A. Spanjaard L. Takumi K.. (2016). Imbalanced presence of *Borrelia burgdorferi* s.l. multilocus sequence types in clinical manifestations of Lyme borreliosis. Infect. Genet. Evol. 42, 66–76. 10.1016/j.meegid.2016.04.019 27125686

[B8] Collares-Pereira M. Couceiro S. Franca I. Kurtenbach K. Schafer S. M. Vitorino L.. (2004). First isolation of *Borrelia lusitaniae* from a human patient. J. Clin. Microbiol. 42, 1316–1318. 10.1128/JCM.42.3.1316-1318.2004 15004107 PMC356816

[B9] Cook M. J. (2015). Lyme borreliosis: a review of data on transmission time after tick attachment. Int. J. Gen. Med. 8, 1–8. 10.2147/IJGM.S73791 25565881 PMC4278789

[B10] Cotté V. Sabatier L. Schnell G. Carmi-Leroy A. Rousselle J. C. Arsene-Ploetze F.. (2014). Differential expression of *Ixodes ricinus* salivary gland proteins in the presence of the *Borrelia burgdorferi* sensu lato complex. J. Proteomics 96, 29–43. 10.1016/j.jprot.2013.10.033 24189444

[B11] des Vignes F. Piesman J. Heffernan R. Schulze T. L. Stafford K. C. III Fish D. (2001). Effect of tick removal on transmission of *Borrelia burgdorferi* and *Ehrlichia phagocytophila* by *Ixodes scapularis* nymphs. J. Infect. Dis. 183, 773–778. 10.1086/318818 11181154

[B12] Eisen L. (2018). Pathogen transmission in relation to duration of attachment by Ixodes scapularis ticks. Ticks Tick Borne Dis. 9, 535–542. 10.1016/j.ttbdis.2018.01.002 29398603 PMC5857464

[B13] Fingerle V. Goettner G. Gern L. Wilske B. Schulte-Spechtel U. (2007). Complementation of a *Borrelia afzelii* OspC mutant highlights the crucial role of OspC for dissemination of *Borrelia afzelii* in *Ixodes ricinus*. Int. J. Med. Microbiol. 297, 97–107. 10.1016/j.ijmm.2006.11.003 17267282

[B14] Fraser C. M. Casjens S. Huang W. M. Sutton G. G. Clayton R. Lathigra R.. (1997). Genomic sequence of a Lyme disease spirochaete, *Borrelia burgdorferi*. Nature 390, 580–586. 10.1038/37551 9403685

[B15] Gern L. Schaible U. E. Simon M. M. (1993). Mode of inoculation of the Lyme disease agent *Borrelia burgdorferi* influences infection and immune responses in inbred strains of mice. J. Infect. Dis. 167, 971–975. 8450262 10.1093/infdis/167.4.971

[B16] Grimm D. Tilly K. Byram R. Stewart P. E. Krum J. G. Bueschel D. M.. (2004). Outer-surface protein C of the Lyme disease spirochete: a protein induced in ticks for infection of mammals. Proc. Natl. Acad. Sci. U.S.A. 101, 3142–3147. 10.1073/pnas.0306845101 14970347 PMC365757

[B17] Hynote E. D. Mervine P. C. Stricker R. B. (2012). Clinical evidence for rapid transmission of Lyme disease following a tickbite. Diagn. Microbiol. Infect. Dis. 72, 188–192. 10.1016/j.diagmicrobio.2011.10.003 22104184

[B18] Kahl O. Janetzki-Mittmann C. Gray J. S. Jonas R. Stein J. de Boer R. (1998). Risk of infection with *Borrelia burgdorferi* sensu lato for a host in relation to the duration of nymphal *Ixodes ricinus* feeding and the method of tick removal. Zentralbl. Bakteriol. 287, 41–52. 9532263 10.1016/s0934-8840(98)80142-4

[B19] Kenedy M. R. Lenhart T. R. Akins D. R. (2012). The role of *Borrelia burgdorferi* outer surface proteins. FEMS Immunol. Med. Microbiol. 66, 1–19. 10.1111/j.1574-695X.2012.00980.x 22540535 PMC3424381

[B20] Koedel U. Fingerle V. Pfister H. W. (2015). Lyme neuroborreliosis-epidemiology, diagnosis and management. Nat. Rev. Neurol. 11, 446–456. 10.1038/nrneurol.2015.121 26215621

[B21] Labuda M. Nuttall P. A. (2004). Tick-borne viruses. Parasitology 129, S221–S245. 10.1017/S0031182004005220 15938513

[B22] Lagal V. Postic D. Ruzic-Sabljic E. Baranton G. (2003). Genetic diversity among *Borrelia* strains determined by single-strand conformation polymorphism analysis of the ospC gene and its association with invasiveness. J. Clin. Microbiol. 41, 5059–5065. 10.1128/jcm.41.11.5059-5065.2003 14605139 PMC262544

[B23] Lindgren E. Jaenson T. G. (2006). Lyme Borreliosis in Europe: Influences of Climate and Climate Change, Epidemiology, Ecology and Adaptation Measures. Copenhagen: WHO Regional Office for Europe.

[B24] Margos G. Vollmer S. A. Cornet M. Garnier M. Fingerle V. Wilske B.. (2009). A new *Borrelia* species defined by multilocus sequence analysis of housekeeping genes. Appl. Environ. Microbiol. 75, 5410–5416. 10.1128/aem.00116-09 19542332 PMC2725479

[B25] Mbow M. L. Christe M. Rutti B. Brossard M. (1994). Absence of acquired resistance to nymphal *Ixodes ricinus* ticks in BALB/c mice developing cutaneous reactions. J. Parasitol. 80, 81–87. 8308662

[B26] Moskvitina G. G. Korenberg E. I. Gorban L. (1995a). [The presence of *Borrelia* in the intestines and salivary glands of spontaneously infected adult *Ixodes persulcatus* Schulze ticks during bloodsucking]. Med. Parazitol. 3, 16–20. 7476674

[B27] Moskvitina G. G. Korenberg E. I. Spielman A. Shchegoleva T. V. (1995b). [The frequency of generalized infection in adult fasting ticks of the genus *Ixodes* in foci of borreliosis in Russia and the USA]. Parazitologiia 29, 353–360. 8524615

[B28] Narasimhan S. Sukumaran B. Bozdogan U. Thomas V. Liang X. DePonte K.. (2007). A tick antioxidant facilitates the Lyme disease agent's successful migration from the mammalian host to the arthropod vector. Cell. Host. Microbe 2, 7–18. 10.1016/j.chom.2007.06.001 18005713 PMC2699493

[B29] Pachner A. R. Itano A. (1990). *Borrelia burgdorferi* infection of the brain: characterization of the organism and response to antibiotics and immune sera in the mouse model. Neurology 40, 1535–1540. 2215944 10.1212/wnl.40.10.1535

[B30] Piesman J. Mather T. N. Sinsky R. J. Spielman A. (1987). Duration of tick attachment and *Borrelia burgdorferi* transmission. J. Clin. Microbiol. 25, 557–558. 3571459 10.1128/jcm.25.3.557-558.1987 PMC265989

[B31] Ramamoorthi N. Narasimhan S. Pal U. Bao F. Yang X. F. Fish D.. (2005). The Lyme disease agent exploits a tick protein to infect the mammalian host. Nature 436, 573–577. 10.1038/nature03812 16049492 PMC4306560

[B32] Ribeiro J. M. Mather T. N. Piesman J. Spielman A. (1987). Dissemination and salivary delivery of Lyme disease spirochetes in vector ticks (*Acari: Ixodidae*). J. Med. Entomol. 24, 201–205. 3585913 10.1093/jmedent/24.2.201

[B33] Rijpkema S. G. Tazelaar D. J. Molkenboer M. J. Noordhoek G. T. Plantinga G. Schouls L. M.. (1997). Detection of *Borrelia afzelii, Borrelia burgdorferi* sensu stricto, *Borrelia garinii* and group VS116 by PCR in skin biopsies of patients with erythema migrans and acrodermatitis chronica atrophicans. Clin. Microbiol. Infect. 3, 109–116. 11864084 10.1111/j.1469-0691.1997.tb00259.x

[B34] Rudenko N. Golovchenko M. Grubhoffer L. Oliver J. H. Jr. (2011). Updates on *Borrelia burgdorferi* sensu lato complex with respect to public health. Ticks Tick Borne Dis. 2, 123–128. 10.1016/j.ttbdis.2011.04.002 21890064 PMC3167092

[B35] Schwan T. G. Burgdorfer W. Schrumpf M. E. Karstens R. H. (1988). The urinary bladder, a consistent source of *Borrelia burgdorferi* in experimentally infected white-footed mice (*Peromyscus leucopus*). J. Clin. Microbiol. 26, 893–895. 3290239 10.1128/jcm.26.5.893-895.1988 PMC266481

[B36] Schwartz I. Wormser G. P. Schwartz J. J. Cooper D. Weissensee P. Gazumyan A.. (1992). Diagnosis of early Lyme disease by polymerase chain reaction amplification and culture of skin biopsies from erythema migrans lesions. J. Clin. Microbiol. 30, 3082–3088. 1452688 10.1128/jcm.30.12.3082-3088.1992 PMC270592

[B37] Šimo L. Kazimirova M. Richardson J. Bonnet S. I. (2017). The Essential Role of Tick Salivary Glands and Saliva in Tick Feeding and Pathogen Transmission. Front. Cell. Infect. Microbiol. 7:281. 10.3389/fcimb.2017.00281 28690983 PMC5479950

[B38] Skotarczak B. (2009). Adaptation factors of *Borrelia* for host and vector. Ann. Agric. Environ. Med. 16, 1–8. 19572471

[B39] Stanek G. Fingerle V. Hunfeld K. P. Jaulhac B. Kaiser R. Krause A.. (2011). Lyme borreliosis: clinical case definitions for diagnosis and management in Europe. Clin. Microbiol. Infect. 17, 69–79. 10.1111/j.1469-0691.2010.03175.x 20132258

[B40] Stanek G. Reiter M. (2011). The expanding Lyme *Borrelia* complex–clinical significance of genomic species? Clin. Microbiol. Infect. 17, 487–493. 10.1111/j.1469-0691.2011.03492.x 21414082

[B41] Stewart P. E. Wang X. Bueschel D. M. Clifton D. R. Grimm D. Tilly K.. (2006). Delineating the requirement for the *Borrelia burgdorferi* virulence factor OspC in the mammalian host. Infect. Immun. 74, 3547–3553. 10.1128/iai.00158-06 16714587 PMC1479289

[B42] Tilly K. Rosa P. A. Stewart P. E. (2008). Biology of infection with *Borrelia burgdorferi*. Infect. Dis. Clin. North Am. 22, 217–234. 10.1016/j.idc.2007.12.013 18452798 PMC2440571

[B43] Vandenesch A. Turbelin C. Couturier E. Arena C. Jaulhac B. Ferquel E.. (2014). Incidence and hospitalisation rates of Lyme borreliosis, France, 2004 to 2012. Euro. Surveill. 19:20883. 10.2807/1560-7917.ES2014.19.34.20883 25188613

[B44] Wikel S. (2013). Ticks and tick-borne pathogens at the cutaneous interface: host defenses, tick countermeasures, and a suitable environment for pathogen establishment. Front. Microbiol. 4:337. 10.3389/fmicb.2013.00337 24312085 PMC3833115

[B45] Wu Q. Liu Z. Wang J. Li Y. Guan G. Yang J.. (2013). Pathogenic analysis of *Borrelia garinii* strain SZ isolated from Northeastern China. Parasit. Vectors 6:177. 10.1186/1756-3305-6-177 23773815 PMC3689080

[B46] Zung J. L. Lewengrub S. Rudzinska M. A. Spielman A. Telford S. R. Piesman J. (1989). Fine structural evidence for the penetration of the Lyme disease spirochete *Borrelia burgdorferi* through the gut and salivary tissues of *Ixodes dammini*. Can. J. Zool. 67, 1737–1748.

